# Global Trade Pattern of Traditional Chinese Medicines and China's Trade Position

**DOI:** 10.3389/fpubh.2022.865887

**Published:** 2022-04-28

**Authors:** Liyao Xiang, Zaoyu Chen, Shaobin Wei, Haiyan Zhou

**Affiliations:** ^1^School of Business Administration, Zhejiang University of Finance and Economics, Hangzhou, China; ^2^School of Economics and Management, Tongji University, Shanghai, China; ^3^Institute of Spatial Planning and Design, Zhejiang University City College, Hangzhou, China; ^4^Institute of International Business and Economics Innovation and Governance, Shanghai University of International Business and Economics, Shanghai, China

**Keywords:** trade of traditional Chinese medicine products, complex network, topological structure, spatial-temporal pattern, interdependence

## Abstract

To depict the evolution of the global trade of traditional Chinese medicine (TCM) products, this article analyzes the 2001–2020 trade data of TCM products in the World Bank and United Nations Commodity Trade Statistics Database to discern the spatial-temporal evolution characteristics of global and China's trade patterns of TCM products from 2001 to 2020 and thereby assess the changes in the global trade of TCM products and in the positions of various countries or regions in the global trade of TCM products. Research findings are as follows: First, on the whole, the total trade volume of TCM products and the number of participating economies and trade connections are on the rise. Second, in terms of topological structure, with higher network density and rising transmission efficiency, the global trade network of TCM products has typical small-world and scale-free network characteristics and has begun to be controlled by a few countries. Judging from the co-opetition between major trading countries, there are more diversified sources of imports for major trading countries, and there is competition between supplying countries. Third, For China, the trade volume of TCM products between China and various countries worldwide has grown rapidly and exhibits a trend of continuous increase followed by decline. China has established extensive trade partnerships and its position in the global trade network of TCM products has been continuously improved. China's participation has contributed to a closer connection among trading entities, but its network heterogeneity remains to be further improved. From the perspective of trade interdependence, the number of countries or regions maintaining high interdependence with China has been gradually increasing, and most of them are European and American countries, Japan, and Southeast Asian countries. The number of countries or regions maintaining low interdependence with China has gradually decreased, and countries or regions that are completely one-way dependent on China are nonexistent.

## Introduction

Traditional Chinese medicines (TCMs) are plants, animals, minerals, and their processed products used to prevent and treat human diseases based on the TCM theory. Conventional TCM products include TCM extracts, health foods, cosmetics, and daily necessities. In 2019, the total volume of TCM commodity imports and exports in China reached US$6.174 billion. Specifically, total exports were worth US$4.019 billion, up to 2.8% YoY; and total imports were worth US$2.155 billion, up to 15.9% YoY. With the deepening of China's opening up, the TCM industry has become a key driver of sustained development of China's health economy. Meanwhile, with dual attributes of science and technology and people's livelihood, the TCM industry is closely related to people's life safety and health and has gradually become a high technology industry that is being competitively developed across the world.

Due to the COVID-19 pandemic, the reconstruction of TCM industry chain has attracted unprecedented attention from the world. The main reason for such attention is the efficacy and economy of TCM products. During the combat against the COVID-19 virus, many TCM products were included in the sixth and seventh protocols for diagnosis and treatment released by China and showed satisfactory clinical effects. The mortality rate of the COVID-19 pandemic has been reduced effectively by the combination of traditional Chinese and western medicine.

According to the statistics from United Nations Commodity Trade Statistics Database, in terms of the total global trade volume of TCM products since 2009, the volatility rose and the average annual growth rate fell to 5.80%. The change in total trade volume is the combined outcome of changes in participating economies and trading volume and shows the increasing complexity of global TCM trade network. However, there are no studies directly discussing the current status of the global TCM trade network and its influencing factors. The following questions exist: Does the TCM trade network possess small-world and power-law characteristics? How will the complex game relations among countries or regions change amid the evolution of the global trade network dominated by major TCM trading countries? Is the relationship between supplying countries necessarily competitive? Ignoring these questions will hinder various countries' adjustment to their TCM industry development strategies and lower the industry's technical innovation efficiency and product quality. Therefore, this article analyzes the international trade network of TCM industry and its influencing factors in an attempt to answer the following questions: First, what changes have occurred to the international trade structure of TCM products? Second, what changes have occurred to the topological structure of the international TCM trade network? Does it have small-world and scale-free characteristics? What is the co-opetition between countries? What are the distribution and spatial patterns of trade groups like?

Considering the above questions, this article first sets up the global TCM trade network and analyzes the network's structural characteristics from the aspects of network connectivity and centrality. Then, with 2001 and 2020 selected as time nodes, the community network analysis method is adopted to look into the evolution characteristics of TCM trade groups and influencing factors of the formation of those groups. Finally, the co-opetition between major TCM trading countries is studied in terms of supply and demand to achieve a deeper understanding of the functionality and structure of the international TCM trade network and provide management implications for countries or regions in the TCM trade network.

### Theoretical Basis

The international trade network is a complex economic system composed of interconnected economies and thus becomes a hot issue in the field of international trade. Based on the literature review, it is found that trade network has been studied from different disciplines and perspectives. In early research, the binary network was mainly adopted for the topology analysis of trade networks. Serrano and Boguna (1) built the trade network employing the complex network analysis method and identified the “small-world,” “scale-free,” and advanced network characteristics of the global trade network by building a trade network. Newman and Park (2) discovered the trade network's topological characteristics, such as betweenness and shortest path length. To gain a deeper insight into the evolution characteristic of the global trade network over time, Mahutga (3), Fagiolo et al. (4, 5), and Xi et al. (6) examined long-term evolution characteristics and arrived at the core-periphery structure, trade cluster, and other conclusions about economies in global trade. With the deepening of academic research, the study of trade networks was divided into the study of industries and the study of products, e.g., the trade of agricultural products and the energy industry (7–9). In addition, a number of scholars analyzed trade networks from regional perspectives, such as the Belt and Road region and Asia-Pacific Economic Region (10, 11).

Based on the review of literature, trade network has become the forefront of theoretical research of social network. Previous studies investigated the characteristics and patterns of trade networks by constructing a binary matrix or weighted directed network, which provides valuable insights for this article. However, the authors also find that further research is required in this field. First, there are few researchers looking into the pattern of TCM trade network from the perspective of whole network and research findings on changes in China's position are inadequate. Second, data mining work that can cover the entire time scale and evolution process of the global TCM trade network needs to be further expanded. The marginal contribution of this article is as follows: Based on the 2001–2020 global TCM trade data and with “TCM trade network” as the object of study, this article breaks the linear logic and considers both time and space dimensions. Through the construction of the 20-year evolution diagram of global TCM trade network, this article attempts to explore the evolution characteristics of the global TCM trade network and analyze the laws of change of China's position in TCM trade in an objective and comprehensive manner to provide a theoretical basis and decision-making support for China's efforts to cope with the changes in TCM trade pattern and build a TCM trade network system.

## Research Methods and Result Analysis

### Research Methods and Data

First of all, the trends of the trade of TCM products of China and the world are analyzed according to the changes in total trade volume. Second, the social network analysis approach is employed to look into the changes in the indicators of the global trade network of TCM products, including network density, average shortest path length, clustering coefficient, centrality, in-degree and out-degree, closeness centrality, betweenness centrality, and trade network group and thus reveal the evolution characteristics of the global trade network of TCM products.

Based on the existing research [e.g., 2, 9, 10] and for the sake of data integrity, this article analyzes the global trade of TCM products in detail through an empirical study of import and export data of TCM products defined under common Chinese Harmonized System (HS) codes 0507, 0510, 091020, 1211, 130190, 130211, 130219, 253090, and 3301. With countries or regions involved in the trade of TCM products abstracted as nodes, this article selects 100 countries or regions, including Mainland China and the United States, as the objects of the study. Given that the imports and exports of TCM products in these countries or regions in 2001–2020^1^ account for 98.50% of the world's total, relevant data are highly representative. With a 100^*^100 matrix of trade network of TCM products built upon bilateral trade flows of the 100 countries or regions, Ucinet is used to analyze the characteristics of this network and Gephi is adopted for visualization of the trade network of TCM products.

### Evolution Characteristics of General Indicators of Global TCM Trade Network

**Figure 1:**
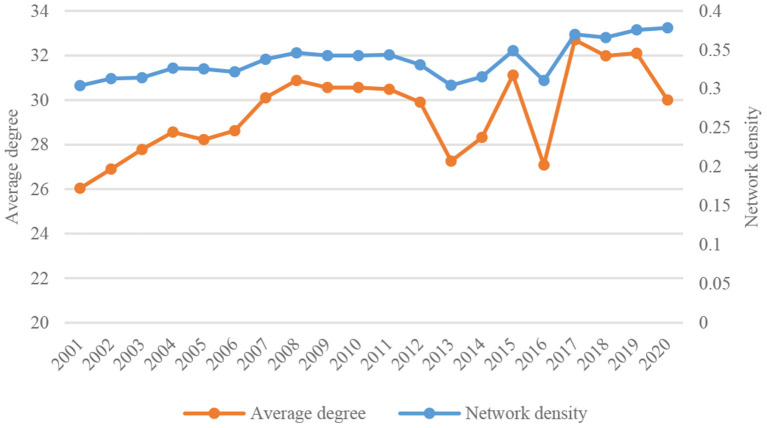
Changes in average degree and density of global TCM trade network in 2001–2020.

**Figure 2:**
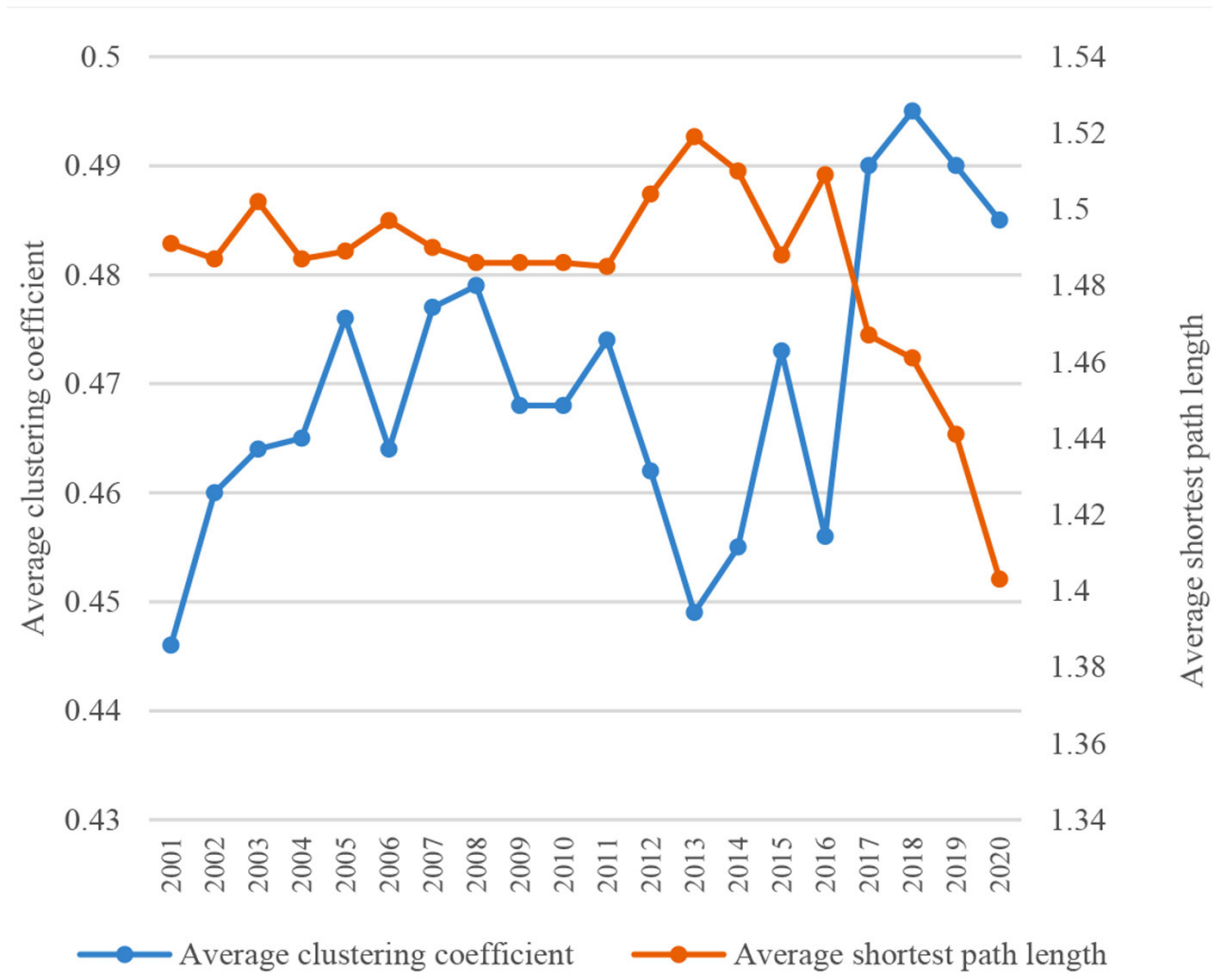
Changes in average clustering coefficient and average shortest path length of global TCM trade network in 2001–2020.

On the whole, the density and transmission efficiency of the global TCM trade network have improved over time. In 2001–2020, the density of the global TCM trade network increased from 0.3042 to 0.3782 and the average degree increased from 26.04 to 30.00 and peaked at 32.70. A denser trade network means a closer relationship among member countries (Figure 1). The average clustering coefficient reflects the clustering degree among countries in the network. The larger the value, the higher the clustering, and vice versa. As shown in Figure 2, the average clustering coefficient of the global TCM trade network ranged between 0.446 and 0.495 and exhibited a general growth trend with fluctuations. A trend of increasing clustering is noticed within the network, especially since 2013. The average shortest path length represents the trade accessibility and network efficiency among member countries. The average path length of the global TCM trade network dropped from 1.491 to 1.403. This means higher connectivity of the network, reduced trade distance between any two economies, and generally improved network transmission efficiency.

The global TCM trade network has typical small-world and scale-free network characteristics. A network with a small average path length and a large clustering coefficient is deemed to have small-world network characteristics. As shown in Figure 2, the change trend of the average path length of the global TCM trade network is contrary to that of the average clustering coefficient. The minimum average shortest path length is 1.487, whereas the maximum clustering coefficient is 0.495. Since the former is about 3 times the latter, the global TCM trade network has typical small-world network characteristics.

**Figure 3:**
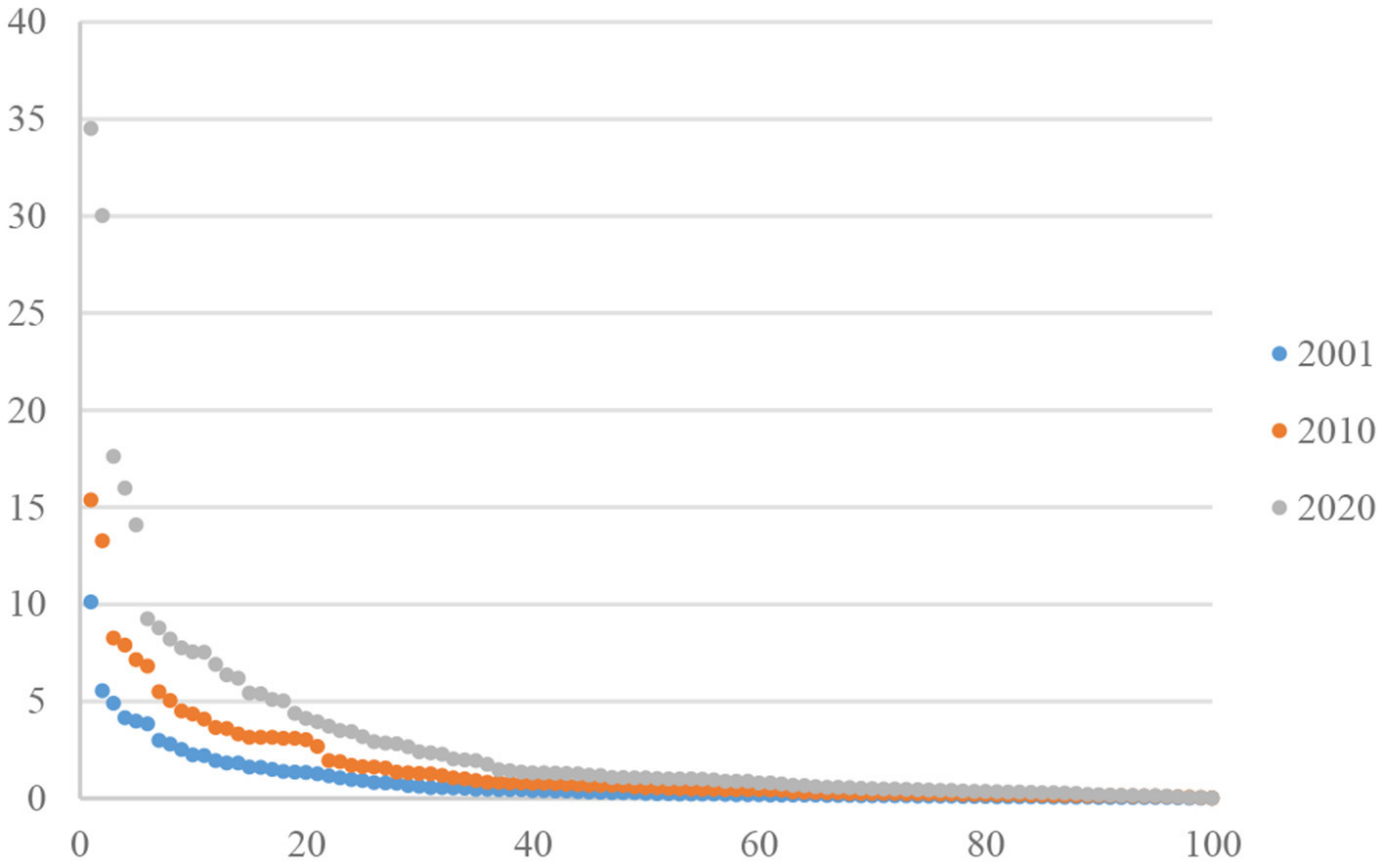
Rank-node degree distribution of global TCM trade network.

Through the comparison among the node degree distribution curves of 2001, 2010, and 2020, the degree distribution curve of 2020 displays a typical long-tailed distribution (Figure 3). Power function fitting is further done on the node degrees of the 3 years. The fitted equation for 2020 is: *y* = 220.19*x*^−1.467^, *R*^2^ = 0.8,051, *p* = 0.001, passing the significance test. This means that the degree follows a power-law distribution. In other words, there were few nodes with high weighted degree and most nodes had low weighted degree in 2020. This follows the scale-free law and indicates that the global TCM trade network began to be controlled by only a few countries.

### Analysis of Core Countries in the Global TCM Trade Network

The weighted degree reflects the position of economies in the global TCM trade market to some extent. All the top 10 countries, except the UK, by weighted degree in 2001 remained in the top 10 in 2020. Specifically, the US ranked first with a weighted degree of 345,061 and China ranked second with 300,060 in 2020. Outdistancing other countries, both the US and China are undisputedly the largest TCM trading countries in the world. However, the global TCM trade pattern changed in 2020. Japan and Hong Kong of China exited from the list of top 5 by weighted degree and the trading position of East Asia generally declined. Meanwhile, India's position rose in the global TCM trade (Table 1).

**Table 1:** Top 10 countries by weighted degree, weighted in-degree, and weighted out-degree in 2001 and 2020.

**2001**						**2020**					
**Country/region**	**C**	**Country/region**	**OD**	**Country/region**	**ID**	**Country/region**	**C**	**Country/region**	**OD**	**Country/region**	**ID**
US	101,026	France	83	France	78	US	345,061	Germany	82	Germany	71
China	55,342	America	77	Germany	74	China	300,060	China	81	China	68
France	48,939	Germany	75	America	68	India	176,016	America	80	US	68
Japan	41,428	UK	75	UK	67	Germany	159,717	India	78	France	66
Hong Kong, China	39,605	India	67	Spain	67	France	140,686	Spain	75	India	65
Germany	38,246	Spain	67	India	61	Spain	92,388	France	73	Spain	64
UK	29,737	Italy	63	Switzerland	60	Japan	87,643	UK	70	UK	61
India	27,864	Japan	59	Italy	56	Republic of Korea	81,881	The Netherlands	64	Italy	59
Spain	25,081	Switzerland	58	The Netherlands	55	The Netherlands	77,393	Egypt	63	The Netherlands	55
South Korea	22,184	The Netherlands	52	China	52	Hong Kong, China	75,370	Switzerland	58	Switzerland	51

Weighted in-degree and weighted out-degree reflect the import and export position of economies in global TCM trade. The list of top 10 countries by weighed out-degree remained relatively stable. France, the US, Germany, the UK, Japan, Switzerland, and The Netherlands stayed in the top 10 for a long time and have long dominated the global TCM export market. In addition, China and Egypt rose quickly into the top 10 rankings by weighted out-degree in 2020. In particular, China ranked only second to the US by weighted out-degree and has become an important node in the global TCM export market. In terms of weighted in-degree, the list of the top 10 countries remained relatively stable. Germany ranked first and China moved to second place in 2020 (Table 1).

Closeness centrality represents the sum of trade connection distances between economies. The higher this value is, the closer a particular economy is to other economies in the trade network. Closeness centrality also indicates the independence and ability to get out of control for a particular node. In terms of closeness centrality (Table 2), the top 10 list in 2001 was dominated by developed European and American countries, including France, Germany, the UK, the US, Spain, Switzerland, Italy, and The Netherlands. The top 10 list in 2020 was the same as that in 2001, except that Switzerland was replaced by Egypt. Moreover, 2/3 of the top 10 countries by closeness centrality were also top 10 countries by in-degree or out-degree. This shows that the global TCM trade network basically follows a peer-to-peer architecture.

**Table 2:** Top 10 countries by closeness centrality and betweenness centrality in 2001 and 2020.

**2001**				**2020**			
**Country/region**	**Closeness**	**Country/region**	**Betweenness**	**Country/region**	**Closeness**	**Country/region**	**Betweenness**
	**centrality**		**centrality**		**centrality**		**centrality**
France	88.889	France	11.565	US	46.919	US	8.091
Germany	83.838	Germany	8.222	China	46.698	China	7.558
UK	79.798	US	6.511	Germany	46.479	France	5.986
US	79.798	UK	5.285	France	45.833	Germany	5.403
Spain	74.747	India	4.819	India	45.833	India	4.993
India	72.727	Spain	4.357	Spain	45.205	Spain	4.722
Switzerland	68.687	Italy	3.13	UK	44.395	UK	2.948
Italy	67.677	China	3.074	The Netherlands	43.805	Egypt	2.753
China	64.646	Switzerland	2.713	Italy	43.231	The Netherlands	2.41
The Netherlands	63.636	Japan	2.297	Egypt	42.857	Italy	1.964

Betweenness centrality is the fraction of international shortest trade connection paths going through a particular economy in the global TCM trade network and reflects the node's control over resources flow. The larger the value, the greater the control. In terms of betweenness centrality (Table 2), France, Germany, the US, the UK, India, Spain, Italy, and China stayed in the top 10 list from 2001 through 2020. Those countries played a vital role and were the major players in the global TCM trade network. Switzerland and Japan declined in their rankings by betweenness centrality, whereas Egypt and The Netherlands rose quickly into the top 10 list. A comparison of the top 10 lists by betweenness centrality, out-degree, and in-degree reveals high similarity, with 8 countries appearing in all the three lists. This shows that the control in TCM trading relationships is directly reflected in the dependence and vulnerability between TCM trading countries.

### Co-opetition Between Major Trading Countries in Global Trade of TCM Products

#### Higher Diversification of Import Sources for Major Trading Countries From the Cooperation Perspective

Trade partners of the top 10 economies in 2020 by weighted in-degree with an import concentration above 5% are selected to build a partial trade network for analysis (Table 3). From the demand-side perspective, major importers of TCM products in 2020 included Germany, China, the US, France, India, Spain, Italy, The Netherlands, and Switzerland.

**Table 3:** Co-opetition between major importers of TCM products in 2020^*a*^.

**Rank**	**Exporter**	**Group**	**Importer**	**Group**	**Concentration**	**Rank**	**Exporter**	**Group**	**Importer**	**Group**	**Concentration**
1	The Netherlands	P1	US	P4	30.95%	29	Germany	P1	Spain	P1	8.18%
2	India	P4	China	P3	27.13%	30	UK	P1	Germany	P1	8.07%
3	US	P4	India	P4	25.25%	31	France	P1	Italy	P1	8.00%
4	Egypt	P4	India	P4	22.52%	32	France	P1	China	P3	7.87%
5	China	P3	US	P4	22.04%	33	France	P1	US	P4	7.75%
6	Egypt	P4	China	P3	21.00%	34	France	P1	Spain	P1	7.62%
7	China	P3	India	P4	19.60%	35	India	P4	US	P4	7.24%
8	Switzerland	P1	US	P4	17.32%	36	Spain	P1	US	P4	7.21%
9	US	P4	China	P3	16.30%	37	Germany	P1	France	P1	7.11%
10	Spain	P1	China	P3	14.92%	38	Switzerland	P1	Italy	P1	7.07%
11	Switzerland	P1	Germany	P1	14.62%	39	Spain	P1	Iran, Islamic Republic of	P3	7.02%
12	Switzerland	P1	France	P1	14.59%	40	India	P4	Vietnam	P3	6.71%
13	UK	P1	US	P4	13.85%	41	The Netherlands	P1	China	P3	6.41%
14	UK	P1	France	P1	13.66%	42	France	P1	Germany	P1	6.35%
15	UK	P1	China	P3	12.23%	43	China	P3	Korea, Republic of	P3	6.35%
16	The Netherlands	P1	Germany	P1	11.81%	44	France	P1	Morocco	P1	6.25%
17	Spain	P1	France	P1	11.71%	45	Spain	P1	Germany	P1	6.09%
18	India	P4	Indonesia	P2	10.98%	46	Egypt	P4	US	P4	6.06%
19	UK	P1	India	P4	9.99%	47	Egypt	P4	Germany	P1	6.04%
20	Germany	P1	India	P4	9.54%	48	Spain	P1	Italy	P1	6.03%
21	France	P1	India	P4	9.40%	49	UK	P1	Spain	P1	6.03%
22	Germany	P1	US	P4	9.39%	50	US	P4	Mexico	P4	5.91%
23	India	P4	Afghanistan	P2	9.27%	51	The Netherlands	P1	Belgium	P1	5.73%
24	Germany	P1	Brazil	P3	8.58%	52	Switzerland	P1	Indonesia	P2	5.50%
25	The Netherlands	P1	France	P1	8.50%	53	The Netherlands	P1	UK	P1	5.21%
26	US	P4	France	P1	8.46%	54	Spain	P1	India	P4	5.03%
27	Egypt	P4	Tunisia	P1	8.36%	55	France	P1	Belgium	P1	5.00%
28	Germany	P1	China	P3	8.36%						

*^a^In 2020, global trade groups of TCM products include: P1 group composed of 14 economies including France, Italy, and Poland, etc.; P2 group composed of 13 economies including United Arab Emirates and Singapore, etc.; P3 group composed of 23 economies including China, Japan, and South Korea, etc.; P4 group composed of 21 economies including the US, Australia, and Canada, etc*.

Within P1, Switzerland's imports from Germany, France, and Italy represent 14.62, 14.59, and 7.07% of its total import of TCM products, respectively. UK's imports from France, Germany, and Spain represent 13.66, 8.07, and 6.03% of its total, respectively. The Netherlands' imports from Germany, France, Belgium, and the UK represent 11.81, 8.50, 5.73, and 5.21% of its total, respectively, and Spain's imports from France, Germany, and Italy represent 11.71, 6.09, and 6.03% of its total, respectively. France's imports from Italy, Spain, Germany, Morocco, and Belgium represent 8.00, 7.62, 6.35, 6.25, and 5.00% of its total, respectively. It is thus clear that Spain and France are important trade partners for each other. By comparison, France had more sources of imports and a low market concentration, whereas Spain had fewer sources of imports and a high market concentration. Within P4, the US and India are important trade partners for each other, and the US, India, and Egypt all have low import concentrations. Outside the groups, P1 and P4 are mainly connected by The Netherlands–US connection. The Netherlands is the largest importer of TCM products from the US, accounting for 30.95% of its total. P3 and P4 are mainly connected by the China–US and China–India connections. P2 and P4 are mainly connected by the India–Indonesia and India–Afghanistan connections. These countries are hubs for the trade of TCM products within and outside the groups.

Among the major purchasing countries or regions of TCM products, China and US had the smallest number of import sources, which were 3 and 4, respectively. India, Egypt, and Switzerland each had 5 sources of imports. Germany and UK each had 6 sources of imports. Spain and France had the largest number of import sources, which were 7 and 8, respectively. In terms of import and export concentration of TCM products, among the top 10 supplying countries, there were 2 countries with an export concentration of >20–30% for their major export partners and 8 countries with 10–20%. By comparison, among the major TCM purchasing countries, there were 3 countries with an import concentration below 10% and 7 countries with 10–20%. In addition, the average number of export partners for TCM supplying countries was 5.3, whereas the 10 major importing countries each had 5.5 sources of imports on average. It is thus evident that the diversification of import sources for major TCM purchasing countries is slightly higher than that of exporters and their import concentration is also slightly lower than the export concentration of major TCM supplying countries. This means importing countries pay more attention to the diversified TCM development strategy.

#### Competition Among All Supplying Countries From the Competition Perspective

The macroscopic expression of co-opetition in the trade of TCM products is the co-opetition between trade groups dominated by major countries. The mesoscopic expression is the trade between countries or regions and the derived co-opetition. Trade partners of the top 10 economies by weighted out-degree in 2020 with an export concentration above 5% are selected to form a partial trade network for analysis (Table 4). From the supply perspective, major exporters of TCM products in 2020 included Germany, China, the US, India, Spain, France, the UK, The Netherlands, Egypt, and Switzerland. Among others, India exported 39.64% of its TCM products to the US, which was its only major export partner. India and China had 3 and 4 major export partners, respectively, to which they exported 18.19 and 13.79% of their total, respectively. The Netherlands, the US, Spain, and the UK each had 5 major export partners, with an export concentration of 11.03, 9.05, 11.81, and 9.74%, respectively. Italy had 6 export partners with an export concentration of 9.84%. As the countries with the highest export diversification, Germany and France each had 7 major export partners, with an export concentration of 7.50 and 9.50%, respectively.

**Table 4:** Co-opetition among major exporters of TCM products in 2020.

**Rank**	**Exporter**	**Group**	**Importer**	**Group**	**Concentration (%)**	**Rank**	**Exporter**	**Group**	**Importer**	**Group**	**Concentration (%)**
1	India	P4	US	P4	39.64	28	Switzerland	P1	Luxembourg	P1	7.52
2	France	P1	US	P4	22.56	29	Germany	P1	Austria	P1	7.23
3	China	P3	US	P4	18.78	30	UK	P1	The Netherlands	P1	7.21
4	Switzerland	P1	US	P4	18.69	31	France	P1	UK	P1	7.08
5	Spain	P1	US	P4	17.66	32	France	P1	Switzerland	P1	7.02
6	Italy	P1	France	P1	17.47	33	Spain	P1	UK	P1	6.99
7	The Netherlands	P1	Germany	P1	17.37	34	Germany	P1	Switzerland	P1	6.90
8	Spain	P1	Germany	P1	16.93	35	UK	P1	France	P1	6.76
9	Italy	P1	US	P4	16.80	36	India	P4	Germany	P1	6.71
10	China	P3	Japan	P3	16.71	37	Germany	P1	The Netherlands	P1	6.63
11	Switzerland	P1	Germany	P1	16.55	38	Germany	P1	France	P1	6.62
12	Germany	P1	US	P4	13.47	39	Switzerland	P1	UK	P1	6.47
13	France	P1	Germany	P1	13.44	40	The Netherlands	P1	US	P4	6.16
14	The Netherlands	P1	Poland	P1	13.43	41	US	P4	Germany	P1	6.13
15	UK	P1	Ireland	P5	12.83	42	Germany	P1	UK	P1	5.92
16	US	P4	Canada	P4	12.82	43	US	P4	Japan	P3	5.79
17	US	P4	China	P3	12.12	44	Germany	P1	Poland	P1	5.71
18	Spain	P1	France	P1	12.05	45	France	P1	The Netherlands	P1	5.62
19	China	P3	Hong Kong, China	P3	11.73	46	Switzerland	P1	Italy	P1	5.56
20	UK	P1	US	P4	11.61	47	Switzerland	P1	France	P1	5.54
21	UK	P1	Germany	P1	10.30	48	France	P1	Italy	P1	5.43
22	The Netherlands	P1	UK	P1	10.19	49	Spain	P1	Italy	P1	5.41
23	Italy	P1	Germany	P1	9.29	50	France	P1	Belgium	P1	5.33
24	US	P4	The Netherlands	P1	8.36	51	Italy	P1	Spain	P1	5.27
25	India	P4	China	P3	8.22	52	Italy	P1	Japan	P3	5.13
26	The Netherlands	P1	Croatia	P1	7.99	53	Italy	P1	UK	P1	5.06
27	China	P3	Korea, Republic of	P3	7.92%						

For TCM supplying countries, the export targets of major supplying countries are the basis of their competitive relationship. Generally speaking, there is competition between supplying countries with the same export direction. Since the foregoing major TCM exporters have the demand to export their TCM products to global markets, there is potential competition among them. Judging from their major export partners, there is competition among all major exporting countries. Both The Netherlands and Switzerland had a large TCM trade with Germany and the US, so there is competition between them. Spain and Italy also had intensive TCM trade relations with those European and American countries. Therefore, there is competition among The Netherlands, Switzerland, Spain, and Italy. Hence, it cannot be generalized whether there is competition between the TCM supplying countries. All in all, there is a competition for European and American markets among The Netherlands, Switzerland, Spain, and Italy.

## Evolution of China'S Trade of TCM Products

### Evolution of Overall Trade Structure Over Time

**Figure 4:**
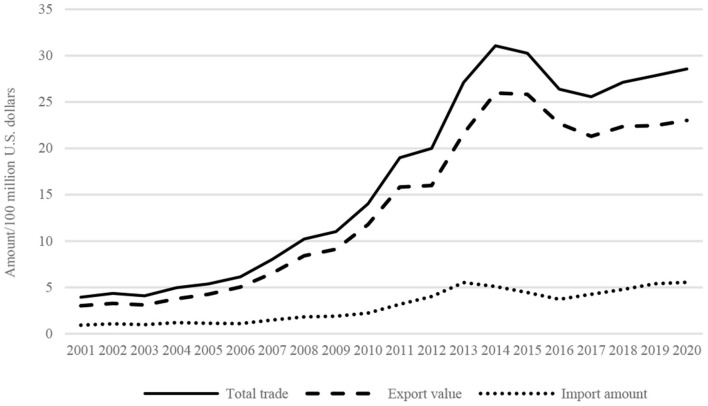
Evolution of China's imports and exports of TCM products in 2001–2020.

The total trade volume of TCM products between China and other countries grew quickly and exhibited a trend of continuous increase followed by decline (Figure 4). The total volume of imports and exports, total exports, and total imports of China with other countries all exhibited a rising trend since China's accession to WTO in 2001, followed by a decline after 2014. The period from 2001 to 2014 was a “golden period” for China's trade of TCM products. The trade volume rose steadily from US$394 million in 2001 to US$3.107 billion in 2014, with an average annual growth rate of 17.89%. After 2014, China's trade volume of TCM products dropped significantly and stood at US$2.557 billion in 2017. Since then, the figure has begun to grow slowly.

### Evolution in Terms of Importers and Exporters

**Figure 5:**
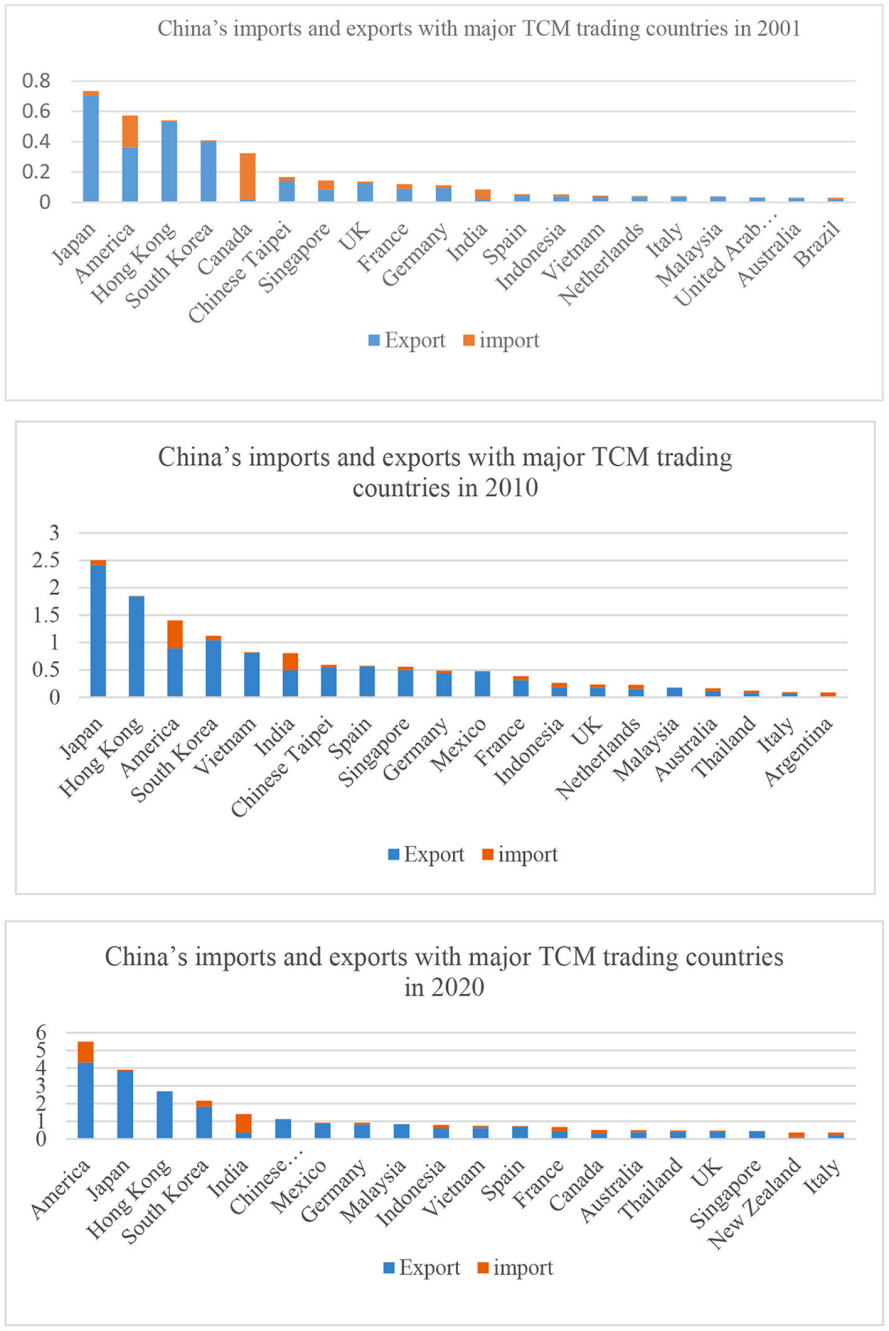
China's imports and exports in 2001, 2010, and 2020.

In terms of imports from major TCM trading countries from 2001 to 2020 (Figure 5), China's imports from Canada have been declining, while thosefrom the US and India have been gradually increasing. Due to China's huge import demand for TCM products from the US and India, they have become China's biggest suppliers of TCM products. For China, other major suppliers of TCM products include South Korea, France, and Canada. In terms of exports to major TCM trading countries in 2001–2020 (Figure 5), the US overtook Japan as China's largest export destination, followed by Japan. Major export destinations of China were mostly East and Southeast Asian countries and developed countries in Europe and America.

### China's Position in Global Trade Network of TCM Products

#### Extensive Trade Partnerships Established by China

**Figure 6:**
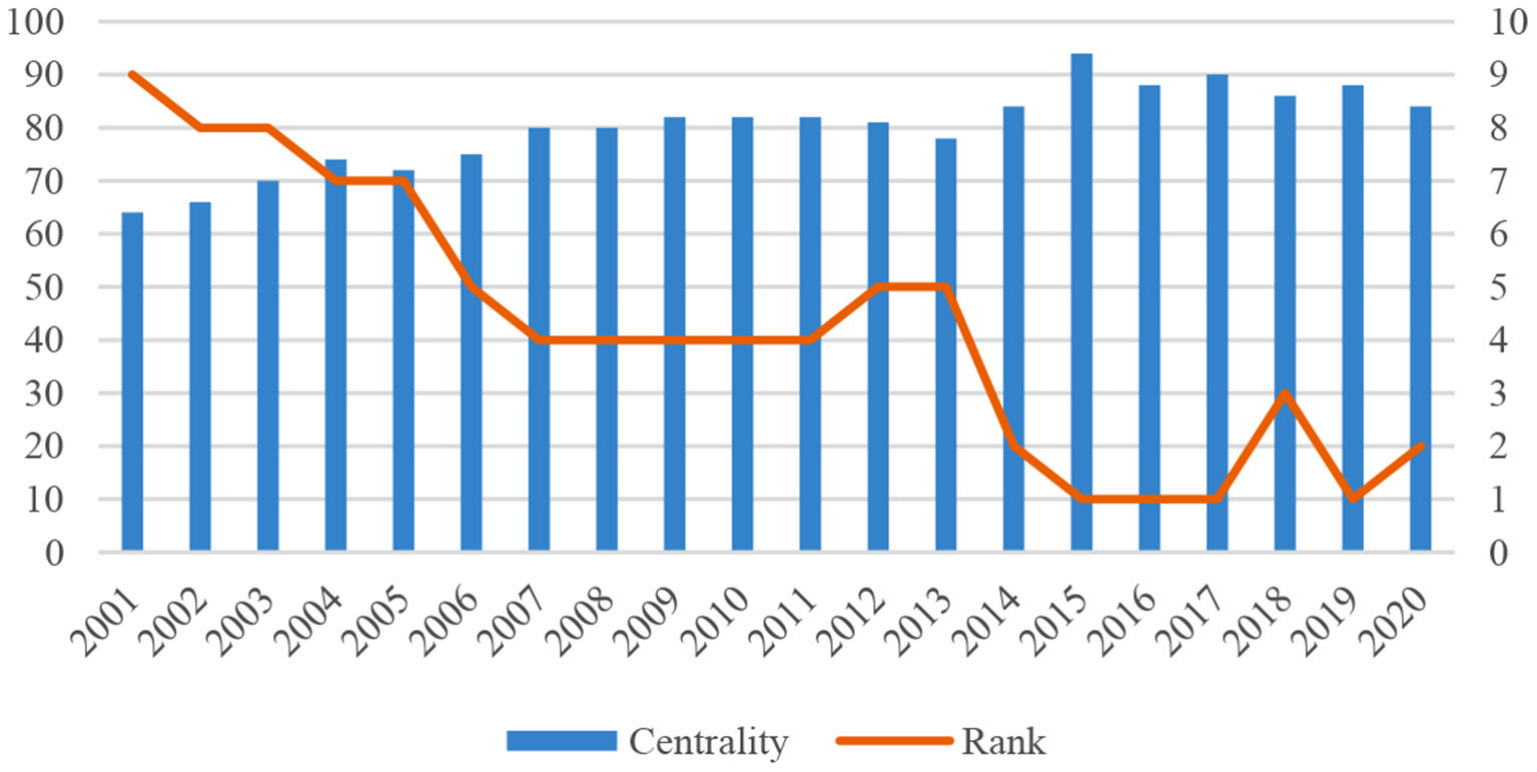
China's network centrality and rank from 2001 to 2020.

The centrality of global trade network of TCM products for China peaked in 2015 when it established trade relations with 99 countries or regions (Figure 6). This means that on the one hand, China has maintained trade relationships with more economies. Its trade partners are more widely distributed in the world and other economies have higher trade dependence on China. On the other hand, China will have larger and more diversified markets and smaller trade risks, suggesting a better position to select its trade partners and optimize its trade structure. It indicates that China occupies a pivotal position in the global trade of TCM products and enjoys a stronger control ability and a higher comparative advantage. China has maintained a core position in the global trade network of TCM products for the past 20 years.

#### China's Trade Network Heterogeneity Needs to Be Improved

**Figure 7:**
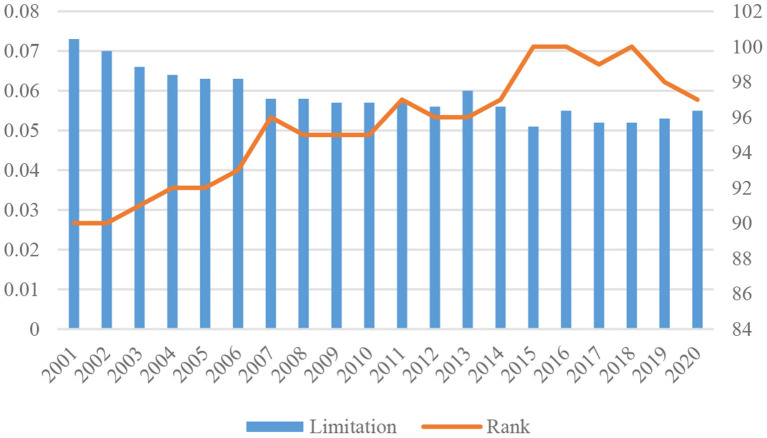
China's structural hole index and rank in 2001–2020.

Figure 7 shows the changes in the ranking of China by structural hole index from 2001 to 2020. In 2001, China ranked 90th with a limitation of 0.073. Afterward, its ranking gradually increased with the declining limitation. In 2007, China rose to 95th with the limitation of 0.058. It ranked 100th in both 2015 and 2016 and ranked 97th in 2020. Obviously, with the declining limitation, China has a stronger ability to occupy structural holes, and its position in the trade network of TCM products has also been continuously improved. Judging from the structural hole index, although China's trade network heterogeneity has improved, it still lags behind other countries. China's trade partnerships are relatively concentrated, thus posing certain trade risks, and its trade network heterogeneity remains to be boosted. China needs to reduce its dependence on existing trade partners by optimizing its geographical and product structures of TCM trade.

#### China's Rising Position in Global Trade Network

**Figure 8:**
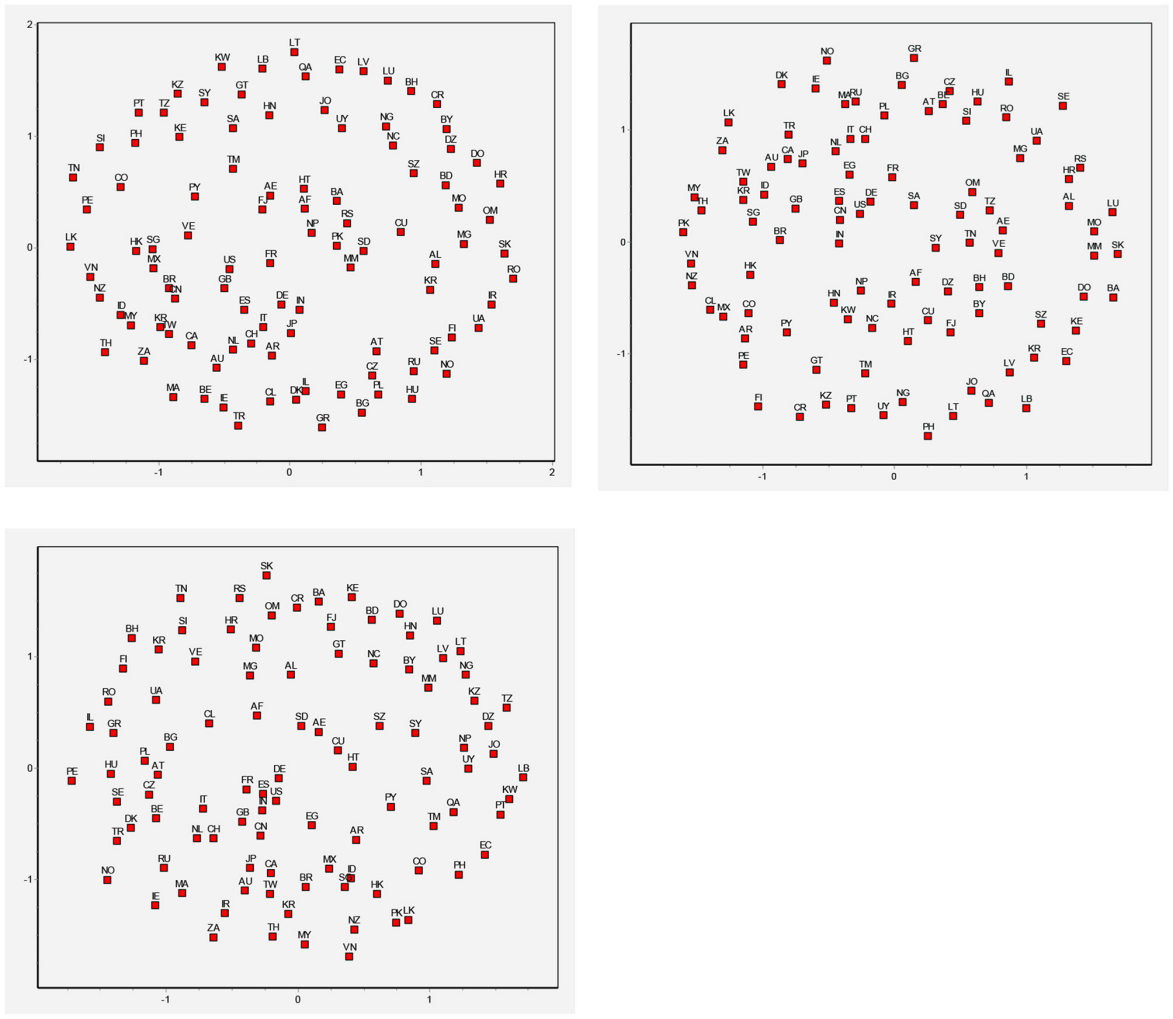
MDS analysis for China in 2001, 2010, and 2020. Afghanistan, AF; Albania, AL; Algeria, DZ; Argentina, AR; Australia, AU; Austria, AT; Bangladesh, BH; Belarus, BD; Belgium, BY; Bosnia and Herzegovina, BE; Brazil, BA; Bulgaria, BR; Chile, BG; Columbia, CA; Costa Rica, CL; Croatia, CN; Cuba, CO; Czech Republic, CR; Denmark, HR; Dominican Republic, CU; Ecuador, CZ; Egypt, DK; Fiji, DO; Finland, EC; France, EG; Germany, SZ; Greece, FJ; Guatemala, FI; Haiti, FR; Honduras, DE; Hungary, GR; India, GT; Indonesia, HT; Ireland, HN; Israel, HK; Italy, HU; Kazakhstan, IN; Kenya, ID; Latvia, IR; Lithuania, IE; Luxemburg, IL; Madagascar, IT; Mexico, JP; Morocco, JO; Nepal, KZ; Netherlands, KE; New Caledonia, KR; Nigeria, KR; Norway, KW; Pakistan, LV; Paraguay, LB; Peru, LT; Philippines, LU; Poland, MO; Portugal, MG; Romania, MY; Russian Federation, MX; Slovakia, MA; Slovenia, MM; North Africa, NP; Spain, NL; Sri Lanka, NC; Sweden, NZ; Switzerland, NG; United Republic of Tanzania, NO; Tunisia, OM; Turkey, PK; Ukraine, PY; United Kingdom, PE; United States of America, PH; Uruguay, PL; Bolivarian Republic of Venezuela, PT; Bahrain, QA; Canada, RO; China, RU; Eswatini, SA; Hong Kong, China, RS; Islamic Republic of Iran, SG; Japan, SK; Jordan, SI; North Korea, ZA; South Korea, ES; Kuwait, LK; Lebanon, SD; Macau, China, SE; Malaysia, CH; Burma, SY; New Zealand, TW; Oman, TZ; Qatar, TH; Saudi Arabia, TN; Serbia, TR; Singapore, TM; Sudan, UA; Syrian Arab Republic, AE; Chinese Taipei, GB; Thailand, US; Turkmenistan (The Mirror), UY; United Arab Emirates, VE; Viet Nam, VN.

Multidimensional scaling (MDS) is a multivariate statistical analysis approach that reflects the correlation between objects in the form of spatial distribution. An MDS map presents an approximate image that visually reflects the interrelations between some high-dimensional sample points in a lower-dimensional space, thus providing information on the relative position of each network node in the trade network and the intimacy of interrelation. The central point of an MDS map represents the central position of the network. The closer a node is to the central point, the closer it is to the center of the network, and the higher its relative position in the network. As shown in Figure 8, Mainland China gradually moved from a semi-periphery position to a central position. Compared with 2001, Mainland China was the closest to the coordinate center in 2020. This indicates that Mainland China plays a vital role in the global trade network of TCM products, which is consistent with the above conclusions obtained with the degree centrality and structural hole index. It can also be found in Figure 8 that China, Japan, South Korea, and the US have remained at the central position of the network for the past 20 years, signifying their high positions in the network.

#### China's Significant Impact on Node Connection in Trade Network

As shown in Table 5, the density of the global trade network of TCM products rose gradually from 2001 to 2020. In 2020, the network density was 1.24 times that in 2001, indicating an increasingly close global trade network of TCM products. Since 2015, China's inclusion in the global trade network of TCM products has improved the network density to different degrees. It indicates that the participation of China has contributed to a closer connection among trading entities.

**Table 5:** China's impact on trade network in 2001–2018.

**Year**	**2001**	**2005**	**2010**	**2015**	**2020**
Density of TCM product trade network	0.3042	0.3256	0.3427	0.3490	0.3782
Density of TCM product trade network without inclusion of China	0.3107	0.3355	0.3476	0.3552	0.3651

### Interdependence of China in the Trade of TCM Products

Based on the foregoing analysis and Figure 5, China's import and export market is similar to that of the world. In terms of trade volume, China's trade of TCM products is highly concentrated. The sum of the import and export volume between China and its top 20 trading partners accounted for more than 85% of its total in 2001, 2010, and 2020, respectively. In terms of spatial distribution, China's import market has been further concentrated in East and Southeast Asian countries, and its export market has begun to be concentrated in European and American countries.

To better portray the interdependence between China and its trading partners in the trade of TCM products, and considering high concentration of China's trade volume of TCM products, this study adopts the independence index model to calculate China's interdependence index with its major trading partners of TCM products in 2001, 2010, and 2020, respectively (Table 6). In terms of the trade of TCM products, the interdependence between China and developed countries or regions, like Canada, Italy, and France, has generally increased, while that with the US and Singapore has decreased significantly. Furthermore, for most European countries or regions with high interdependence with China, China's exports to them were lower than their exports to China, which indicates that China is more dependent on those countries or regions. Meanwhile, China's exports to countries or regions with low interdependence have gradually increased and were much higher than their exports to China, resulting in low interdependence but high one-way dependence.

**Table 6:** Interdependence between China and its top 20 trading partners in 2001, 2010, and 2020.

**Rank**	**2001**		**2010**		**2020**	
	**Country/region**	**DrGL*_***i,j***_***	**Country/region**	**DrGL*_***i,j***_***	**Country/region**	**DrGL*_***i,j***_***
1	Japan	0.08	Japan	0.07	US	0.43
2	US	0.73	Hong Kong, China	0.01	Japan	0.04
3	Hong Kong, China	0.04	US	0.73	Hong Kong, China	0.01
4	South Korea	0.03	South Korea	0.14	Republic of Korea	0.31
5	Canada	0.12	Vietnam	0.04	India	0.51
6	Chinese Taipei	0.29	India	0.78	Chinese Taipei	0.05
7	Singapore	0.84	Chinese Taipei	0.15	Mexico	0.07
8	UK	0.16	Spain	0.05	Germany	0.23
9	France	0.55	Singapore	0.22	Malaysia	0.00
10	Germany	0.32	Germany	0.19	Indonesia	0.51
11	India	0.47	Mexico	0.01	Vietnam	0.34
12	Spain	0.36	France	0.39	Spain	0.10
13	Indonesia	0.47	Indonesia	0.6	France	0.75
14	Vietnam	0.36	UK	0.5	Canada	0.86
15	The Netherlands	0.24	The Netherlands	0.65	Australia	0.48
16	Italy	0.11	Malaysia	0.01	Thailand	0.36
17	Malaysia	0.09	Australia	0.56	UK	0.36
18	United Arab Emirates	0	Thailand	0.66	Singapore	0.07
19	Australia	0.19	Italy	0.43	New Zealand	0.59
20	Brazil	0.68	Argentina	0.13	Italy	0.76

According to the ranking of China's major trading partners by the interdependence index (Table 7), the number of countries or regions that maintain high interdependence with China on the trade of TCM products has gradually increased and they are mostly European and American countries, Japan, and Southeast Asian countries. The number of countries or regions that maintain low interdependence with China has gradually decreased, and the countries or regions that are completely one-way dependent on China are nonexistent.

**Table 7:** Ranking of China's top 20 trading partners in trade of TCM products by interdependence in 2001, 2010, and 2020.

**Interdependence**	**2001**	**2010**	**2020**
High	Singapore, US, Brazil, France	India, US, Thailand, The Netherlands, Indonesia, Australia, UK	Canada, Italy, France, New Zealand, India, Indonesia
Medium	India, Indonesia, Spain, Vietnam, Germany, Chinese Taipei, The Netherlands	Italy, France, Singapore	Australia, US, Thailand, UK, Vietnam, South Korea, Germany
Low	Australia, the UK, Canada, Italy, Malaysia, Japan, Hong Kong (China), South Korea, United Arab Emirates	Germany, Chinese Taipei, South Korea, Argentina, Japan, Spain, Vietnam, Mexico, Malaysia, Hong Kong (China)	Spain, Mexico, Singapore, Chinese Taipei, Japan, Hong Kong (China), Malaysia

On the whole, China has established extensive interdependent relations and almost no one-way dependent relations. Among its major trading partners of TCM products, the interdependence of China with European and American countries, Japan, and Southeast Asian countries has generally deepened.

## Conclusion And Discussion

### Conclusion

To depict the evolution of the global trade of TCM products, this article analyzes the 2001–2020 trade data of TCM products in the World Bank and United Nations Commodity Trade Statistics Database to discern the spatial-temporal evolution characteristics of global and China's trade patterns of TCM products from 2001 to 2020 and thereby assess the changes in global and China's trade of TCM products and in China's position in the global trade of TCM products. The major findings of this study are as follows.

First, the total trade volume of TCM products is generally on the rise. The changes in total trade volume are the combined outcome of changes in participating economies and trade volumes. The development trends of the number of participating economies and trade connections generally correspond to the total trade volume. With increasing interactions and interdependence, TCM trade connections between countries or regions have strengthened.

Second, in terms of topological structure, with higher density and rising transmission efficiency, the global trade network of TCM products has typical small-world and scale-free network characteristics and has begun to be controlled by a few countries. Based on the analysis of core countries, China and the US are undisputedly the largest TCM traders in the world. The global trade network of TCM products basically follows a peer-to-peer architecture. The control in TCM trade is directly reflected in the dependence and vulnerability between TCM trading countries. Judging from co-opetition between major trading countries, there are more diversified import sources for major trading countries, and there is competition between supplying countries.

Third, for China, the trade volume of TCM products between China and various countries worldwide has grown rapidly and exhibits a trend of continuous increase followed by decline. In terms of imports from major trading countries, China's imports from Canada have been declining, while those from the US and India have been gradually increasing. In terms of exports, the major export destinations of China were mostly East and Southeast Asian countries and developed countries in Europe and America. China has established extensive trade partnerships and its position in the global trade network of TCM products has been continuously improved. China's participation has contributed to a closer connection among trading entities, but its network heterogeneity remains to be further improved.

From the perspective of trade interdependence, the number of countries or regions maintaining high interdependence with China has been gradually increasing, and most of them are European and American countries, Japan, and Southeast Asian countries. The number of countries or regions maintaining low interdependence with China has been gradually decreasing, and countries or regions that are completely one-way dependent on China are nonexistent.

### Theoretical Contributions

The global economic situation is currently complicated, and reverse globalization and new trade protectionism are on the rise. The trade of TCM products is also affected. An in-depth analysis of the global TCM trade network is of positive significance to the development of global TCM trade and the quality improvement of China's TCM import and export trade.

In terms of theoretical foundation and hypothesis, previous studies on TCM trade focused more on technology transfer (12), potential estimation (13, 14), and influencing factors (15, 16). Unlike previous studies, this article adopts a multi-year scale approach to discuss the overall structural evolution of global TCM trade from a more macroscopic perspective. Similar to the findings by Hinsley et al. (17) and Cheung et al. (18), this article proceeds from the perspective of complex network and draws conclusions, including scale-free characteristics and shift of focus of import and export in the global trade of TCM products, asserting the existence of dependence and competition among countries or regions. Unlike previous studies, the results of this study show that the global TCM trade network is a typical small-world network that is closely connected and continuously expanding. Major export destinations of China were mostly East and Southeast Asian countries and developed countries in Europe and America. Moreover, this network follows a peer-to-peer architecture. The control in TCM trade relationship is directly reflected in dependence and vulnerability between TCM trading countries.

This article reveals the evolution characteristics of spatial-temporal pattern of global trade of TCM products and explains the changes in the trade network from various perspectives. The conclusions are believed to assist in China's adjustment of its TCM products import and export policies and help China make breakthroughs in the development of emerging markets. This research is also a meaningful exploration for China's communication and cooperation with other countries on TCM products. This research also fills a gap in previous studies by delving into spatial-temporal evolution of TCM trade and for the first time discusses the trade groups, spatial pattern, and evolution of import and export structure of TCM products. This study is a significant contribution from both theoretical and practical perspectives.

On the whole, this article has two new elements compared with existing research findings: First, it provides a new perspective on the TCM trade. Most existing studies look into the current status and trade potential of the TCM trade industry (12, 15), and there are few studies focusing on the spatial-temporal evolution of import and export trade of TCM products, which actually has an important influence on imports and exports of the TCM products industry. Second, this study applies an existing methodology in a new context. This article adopts the social network analysis method used in previous studies (2) to examine the trade network of TCM products. On the one hand, the scope of application of social network analysis approach is expanded. On the other hand, this method enables analyzing the current status of the TCM trade network composed of various trading countries or regions in a relatively scientific manner.

## Data Availability Statement

The original contributions presented in the study are included in the article/supplementary material, further inquiries can be directed to the corresponding author.

## Author Contributions

All authors undertook research, writing, and review tasks throughout this study. All authors have read and agreed to the published version of the manuscript.

## Funding

This work was supported by the Major Program of the National Social Science Foundation of China [Grant Number 20&ZD124], the National Social Science Foundation of China [Grant Number 21CJY024], and the National Natural Science Foundation of China [Grant Numbers 72174180, 72074195, 71973129, 72072162, and 71773115].

## Conflict of Interest

The authors declare that the research was conducted in the absence of any commercial or financial relationships that could be construed as a potential conflict of interest.

## Publisher's Note

All claims expressed in this article are solely those of the authors and do not necessarily represent those of their affiliated organizations, or those of the publisher, the editors and the reviewers. Any product that may be evaluated in this article, or claim that may be made by its manufacturer, is not guaranteed or endorsed by the publisher.

## Footnotes

^1^There are many missing values in the data before 2001. In order to ensure the integrity of the data, we selected the data after 2001 as the sample.
